# A Case of Rickets and Pediatric Iron Deficiency Anemia in Alabama

**DOI:** 10.7759/cureus.60140

**Published:** 2024-05-12

**Authors:** Claudia G Reid, Rhonda Graham

**Affiliations:** 1 Pediatrics, Edward Via College of Osteopathic Medicine, Auburn, USA; 2 Pediatrics, Edward Via College of Osteopathic Medicine, Huntsville, USA

**Keywords:** supplementation guidelines, early nutrition, developmental pediatrics, iron deficiency anemia (ida), nutritional rickets

## Abstract

A 15-month-old African American male patient presented to the pediatric clinic to establish care. The patient had been seen and treated by a previous pediatrician who had diagnosed him with failure to thrive, anemia, and hepatosplenomegaly, according to the patient’s parents. Upon physical examination, the patient was determined to be less than the first percentile for height and in the eighth percentile for weight. Frontal bossing was also observed. The patient’s hemoglobin level was measured in the office to help confirm the previous anemia diagnosis and was determined to be 6.3 g/dL (normal: 10.5-13.0 g/dL). At this point, the patient was sent to a pediatric emergency department for continued treatment and workup. At the emergency department, the patient received an extensive laboratory workup for the evaluation of anemia, revealing iron deficiency anemia (hemoglobin: 5.6 g/dL (normal: 10.5-13 g/dL), mean corpuscular volume: 51.4 fl (normal: 70-84 fl), iron: 18 mcg/dL (normal: 30-70 mcg/dL), total iron binding capacity: 598 mcg/dL (normal: 100-400 mcg/dL), and hematocrit: 23.7% (normal: 33-38%)) and decreased levels of vitamin D (<6 ng/mL, normal: >30 ng/mL), ionized calcium (1.17 mg/dL, normal: 4.4-5.2 mg/dL), and phosphorus (2.4 mg/dL, normal: 2.9-5.9 mg/dL). These studies, paired with X-ray images of the patient’s shoulders and wrists, further confirmed the diagnosis of rickets. Rickets is a disease in pediatric patients defined as a condition in which the mineralization of epiphyseal plates is defective. A nutritional deficiency in vitamin D, calcium, or phosphate causes acquired rickets. This condition is most commonly found in developing countries; some predisposing factors include poor sun exposure, high altitude, and breastfeeding. The patient was seen in the outpatient pediatric setting after the hospitalization, in which he received a blood transfusion, where he was managed on supplementation of calcium carbonate suspension, polysaccharide iron complex/novaferrum drops, and cholecalciferol drops with referral to endocrinology, hematology, and dietetics. This case serves as an example of how the diagnosis of nutritional deficiencies, such as rickets, can also be found in developed countries like the United States. Other conditions considered in the differential diagnosis were cystic fibrosis, necrotizing enterocolitis, metabolic disorders, inadequate absorption, and mechanical feeding difficulties, each of which must be ruled out to ensure that even an unlikely finding was not missed.

## Introduction

Exclusively breastfed infants can be at risk of many nutritional deficiencies, including iron, zinc, vitamins A, D, C, and E, amino acids, and essential fatty acid deficiencies [1]. Breast milk is an ideal infant food source, with benefits like increased mother-child bonding, promotion of biological and immune properties, cost-effectiveness, and decreased infant morbidity and mortality [1]. The first year of an infant’s life is essential for developmental milestones and stages. If nutrition is not prioritized and interventions are not made, there is much potential for threats to human health, including immediate implications related to poor growth and long-term concerns, including delayed neurocognitive development and noncommunicable diseases [1]. With these implications in mind, infants who have a delay in complementary feeding initiation or poor nutritional quality of complementary foods could be at risk for developing conditions such as rickets and iron deficiency anemia (IDA).

Rickets is caused by vitamin D, calcium, and phosphate deficiencies. It is linked to biochemical abnormalities, bone deformities, impaired growth, and developmental delays [2]. Some well-known bone deformities due to rickets include bowing of the legs, knock knees, frontal bossing, widening of the metaphysis of long bones, rachitic rosary, and pathologic fractures in more severe cases. Rickets is a rarer finding in developed countries and more prevalent in developing countries. In a study by Hayat et al., nutritional rickets was present in 36% of breastfed infants, with associated factors including low socioeconomic status and no formal education [2]. With these associated findings, physicians should consider socioeconomic factors and education in their pursuit of helping to prevent nutritional deficiencies, as they are both important determinants of outcomes.

IDA must also be considered for exclusively breastfed infants. Iron stores at birth can meet iron needs for the first few months of life, but when that runs out, breast milk alone does not meet the iron requirements at any age [3]. To prevent IDA, adequate iron intake is necessary and can be accomplished through complementary feeding, especially in the second half of infancy [3,4]. IDA and iron deficiency status can lead to long-term sequelae, including neurodevelopmental and behavioral effects, some of which may be irreversible [5]. According to Rigo and Senterre, it is recommended that preterm infants fed with human milk receive supplementation with iron of 2 mg/kg starting at one month of age up until 12 months of age to help prevent IDA and its associated consequences [6].

This case report describes a 15-month-old infant with nutritional rickets and IDA.

## Case presentation

**Figure 1:**
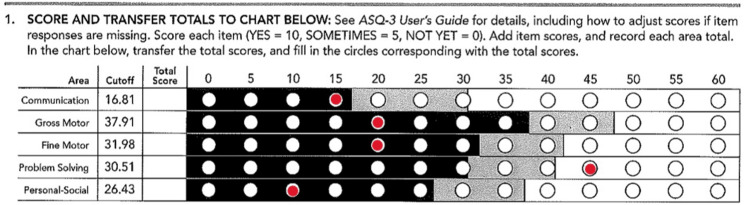
CHADIS Ages & Stages Questionnaire, Third Edition scoring results The red circular markings indicate the patient’s score for each respective field of the questionnaire [7].

A 15-month-old African American male presented to the pediatric clinic with both of his parents to establish care. The patient’s history, as reported by his parents, included a previous diagnosis of failure to thrive, anemia, and hepatosplenomegaly. The patient’s previous medical records were not available during the encounter. The mother of the patient reported that the patient had been thriving and doing well recently while being alert and active. The patient was born at 36 weeks gestation and had a one-week stay in the neonatal intensive care unit after birth. The mother had complications at birth that included gestational diabetes and preeclampsia. The patient was reported to live at home with both parents and two older sisters. The patient was still nursing on demand and mostly only taking breast milk without supplementation with vitamin D or cow’s milk. The patient had been previously introduced to soft foods, including macaroni and cheese, mashed potatoes, rice, fruits, and vegetables. The patient was up to date on all vaccinations, had no previous surgical history, and had no known drug allergies. At this visit, the patient’s mother filled out the CHADIS Ages & Stages Questionnaire, Third Edition (ASQ-3). This questionnaire showed the result of failure in communication, gross motor, fine motor, and personal-social areas. Results from the CHADIS Questionnaire are presented in Figure 1 [7]. During the 15-month visit, the review of systems was all within normal limits.

Upon measurement of vital signs, the patient was determined to be 27.2 inches in length (less than the first percentile for his current age) and 19 pounds and 8.32 ounces in weight (eighth percentile for his current age). The BMI was determined to be 18.5 mg/m^2^. The temperature was 97.8 °F. Frontal bossing and a grade 1 systolic flow murmur were observed on physical examination. No hepatosplenomegaly was noted on examination. During the developmental assessment, the patient was not speaking at all and had not taken any steps yet.

The patient’s hemoglobin level was measured three times in the outpatient pediatric clinic. It was found to be 6.3 g/dL (normal: 10.9-15 g/dL). At that point, the patient was determined to have a diagnosis of anemia of unknown etiology, with further workup needed. Because of the severely low hemoglobin level, the patient’s parents were instructed to take him directly to the closest pediatric emergency department, where an extensive laboratory workup was completed and imaging studies were done.

A detailed outline of the lab results with normal reference ranges of the values from the patient’s hospitalization is outlined in Table 1 [8]. The lab results showed the presence of IDA and rickets caused by decreased levels of vitamin D, calcium, and phosphorus. X-ray studies of the patient’s wrists and shoulders showed widening and fraying of the metaphyses.

**Table 1:** Lab results from hospitalization Normal values for the patient’s age and corresponding units are included in the table [8].

Lab measured	Value	Normal	Units
Sodium	138 mmol/L	136-145 mmol/L	mmol/L
Potassium	4.7 mmol/L	3.5-5.0 mmol/L	mmol/L
Creatinine	0.2 mg/dL	0.5-1.5 mg/dL	mg/dL
White blood cell count	6.19 ×10^3^/mm^3^	6.0-17.0 × 10^3^/mm^3^	mm^3^
Hemoglobin	5.6 g/dL	10.5-13.0 g/dL	g/dL
Alkaline phosphatase	598 units/L	85-400 units/L	units/L
Mean corpuscular volume	54.1 fl	70-84 fl	fl
Iron	18 mcg/dL	30-70 mcg/dL	mcg/dL
Total iron binding capacity	598 mcg/dL	100-400 mcg/dL	mcg/dL
Hematocrit	23.70%	33-38%	%
Red cell distribution width	24.40%	<16%	%
Vitamin D	<6 ng/mL	>30 ng/mL	ng/mL
Ionized calcium	1.17 mg/dL	4.4-5.4 mg/dL	mg/dL
Parathyroid hormone	114 pg/mL	11-59 pg/mL	pg/mL
Phosphorous	2.4 mg/dL	2.9-5.9 mg/dL	mg/dL
Magnesium	1.12 mg/dL	1.5-2.5 mg/dL	mg/dL

The patient was given a blood transfusion while in the emergency department. The patient was also prescribed calcium carbonate suspension, polysaccharide iron complex/nevoferrum drops, and cholecalciferol drops at the hospital. The patient was additionally referred to hematology, endocrinology, and dietetics.

The patient was seen again in the pediatric clinic the following week, after hospitalization. Diagnoses at that time included IDA, rickets, failure to thrive, and developmental delay. The patient was directed to continue all supplementation prescribed during hospitalization and to keep pending appointments with endocrinology, hematology, and dietetics. Due to the developmental delays, the patient was referred to speech therapy and occupational therapy. The patient was scheduled to be seen again for an 18-month appointment for further evaluation.

## Discussion

In the case report described, the patient was diagnosed and treated for nutritional rickets and IDA at 15 months of age due to exclusive breastfeeding without supplementation since birth. Without any supplementation while breastfeeding, the patient developed several nutritional deficiencies, including calcium, phosphate, vitamin D, and iron. Upon examination, the patient demonstrated indications for a diagnosis of failure to thrive and several developmental delays.

These nutritional deficiencies could have been prevented or had a presentation with decreased severity if the patient had been closely followed up after birth and nutrition recommendations had been presented earlier. Breastfed infants are at risk of developing vitamin D deficiency due to the low concentration of vitamin D found in breast milk [9]. The American Academy of Pediatrics calls for supplementation with vitamin D at 400 IU/d beginning soon after birth in breastfed infants [10]. Maternal supplementation with vitamin D to enrich breast milk for infants is another method that has been studied to help meet infants’ vitamin D requirements [11]. Calcium consumption in an infant depends on the mother’s diet in breastfed infants; thus, calcium supplementation should also be considered on a case-by-case basis depending on how much calcium the mother of the infant consumes in her daily diet [12]. To help prevent IDA in infants, it is recommended that cow’s milk be avoided in the first year of life, that iron supplementation begin at four to six months in breastfed infants, and that formula-fed infants use iron-fortified formula [13]. Iron supplementation, if indicated, should provide 1 mg of elemental iron per kilogram per day [13]. Oral ferrous sulfate drops are another option for supplementation and can contain 10 mg of elemental iron per mL [14].

Educating parents on the importance of infant nutrition to prevent deficiencies with the above recommendations is essential during regular follow-up visits for infants. Infancy is crucial for physical and cognitive development, and consequences can be avoided with attention to detail and awareness. Socioeconomic status and ability to obtain nutrients should also be considered during follow-up visits with infants, as nutritional rickets are more prevalent in lower-income environments [15]. In similar cases, multidisciplinary teams that include pediatricians, social workers, developmental and behavioral professionals, dietitians, hematologists, and endocrinologists should be included to ensure that the patient receives well-rounded care and that all aspects of the patient’s condition are managed appropriately.

Additionally, physicians should keep the diagnosis of rickets in mind while developing a differential diagnosis when a patient presents with failure to thrive, developmental delays, and orthopedic abnormalities [16]. Rickets is generally known to be more common in developing countries or a disease of the past in the United States, but in 2013, the case rate of rickets was found to be 2.9-27 per 100,000 patients in the United States and Europe [17]. With the increasing occurrence of this condition, physicians should be vigilant not to miss this diagnosis.

## Conclusions

Early education, supplementation, and consistent and vigilant follow-up starting from birth can help prevent deficiencies that may result from being exclusively breastfed for prolonged infancy. This can further prevent additional sequelae, including developmental and growth delays, associated with the nutritional deficiencies of rickets and IDA.
